# Ultrasound-assisted synthesis of new bisphosphonate–betulin conjugates and preliminary evaluation of their cytotoxic activity†

**DOI:** 10.1039/d4ra07782b

**Published:** 2025-02-06

**Authors:** Dominika Kozicka, Małgorzata Krześniak, Mirosława Grymel, Jakub Adamek, Barbara Łasut-Szyszka, Tomasz Cichoń, Anna Kuźnik

**Affiliations:** a Department of Organic Chemistry, Bioorganic Chemistry and Biotechnology, Silesian University of Technology B. Krzywoustego 4 44-100 Gliwice Poland anna.kuznik@polsl.pl; b Biotechnology Center, Silesian University of Technology B. Krzywoustego 8 44-100 Gliwice Poland; c Center for Translational Research and Molecular Biology of Cancer, Maria Skłodowska-Curie National Research Institute of Oncology, Gliwice Branch Wybrzeże Armii Krajowej 15 44-102 Gliwice Poland

## Abstract

Bisphosphonates (BPs) are a well-established group of drugs that have been used for decades in the prevention and treatment of osteoporosis and cancer treatment-induced bone loss. Their unique properties such as high bone affinity, enzymatic stability as well as a multidirectional biological activity prompt the creation of BP conjugates. In this study, we designed and synthesized three new bisphosphonate conjugates with betulin, a natural product with a high safety profile and a broad spectrum of biological activity. The designed conjugates differed in the type of linker used and the number of bisphosphonate moieties attached (mono- or disubstituted derivatives). The proposed method for their synthesis proceeds under mild reaction conditions and gives good yields of products. In addition, as we have shown, the reaction can be assisted by ultrasound, which significantly reduced the reaction time (from 48 hours to 2 hours) and improved the overall product yield (up to 92%). The cytotoxicity of the new conjugates was evaluated against osteosarcoma (U-2 OS), lung adenocarcinoma (A549) and gastric adenocarcinoma (AGS) cell lines. The results of preliminary biological studies showed that the obtained conjugates had improved solubility compared to that of betulin and exhibited a cytotoxic effect on all three tested cell lines at the micromolar level. The betulin analog having two bisphosphonate groups 6 demonstrated the highest cytotoxic activity against tested cell lines (IC50 between 5.16 and 6.21 μM).

## Abstract 2

A protocol for the ultrasound-assisted synthesis of new biologically active betulin–bisphosphonate conjugates in friendly reaction conditions was reported.

## Graphical abstract

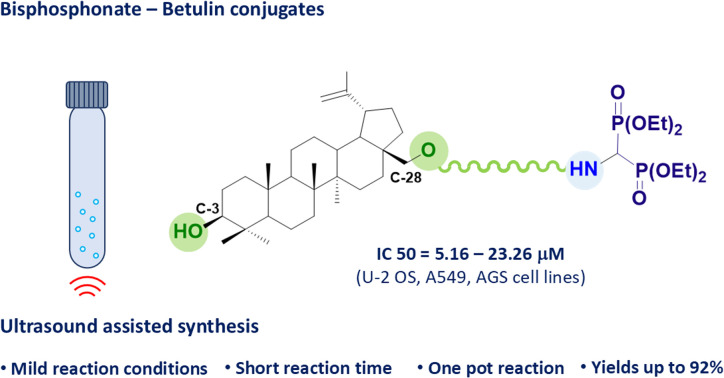

## Introduction

Bisphosphonates (BPs) are synthetic analogs of naturally occurring inorganic pyrophosphates, characterized by the presence of a P–C–P skeleton.^1^ Although they are primarily known as antiresorptive drugs, they exhibit a much broader spectrum of biological activity, including antibacterial,^2^ antiviral,^3–5^ antiparasitic^6,7^ and anticancer activities.^8^ Both *in vitro* and *in vivo* studies involving newer-generation bisphosphonates (zoledronate, rizedronate, ibandronate) have shown that they inhibit tumor cell proliferation, induce apoptosis, activate the immune system to increase lymphocyte cytotoxicity γδT,^8,9^ and inhibit the growth of cervical cancer,^10^ mesothelioma,^11^ osteosarcoma tumor cells,^12,13^ and bone metastasis.^14,15^ Despite their cytotoxic effect, bisphosphonates are used in cancer therapies as adjuvants to inhibit the occurrence of skeletal-related events (SREs) or reduce bone pain, rather than as cytostatics. Satisfactory effects and a visible therapeutic effect, such as reduction of tumor growth or inhibition of metastases to soft tissues, were observed at very high BP concentrations.^16^ This can be attributed to the low bioavailability of bisphosphonate acids, caused by their high ionization at physiological pH values.^17^

In addition, bisphosphonates also exhibit a high affinity for bones due to the ability of phosphonic acid groups to interact with hydroxyapatite (HAP), the main component of bones.^18^ Although BPs' binding affinity has contributed to their tremendous therapeutic success as antiresorptive drugs, it prevents their other useful properties from being used to treat non-skeletal diseases. However, BPs' bone binding ability is dependent on their structure and can be modulated by the degree of esterification of the phosphonic groups. As Puljula *et al.* have shown, simple BPs (etidronate, medronate) with three ester groups no longer have an affinity for HAP.^19^ Furthermore, simple dialkyl esters of bisphosphonates (*e.g.* dimethyl) are stable *in vivo*, and thus their chelating effect is permanently masked.^20,21^ Esterification of the phosphonic groups not only reduces bone affinity but also increases lipophilicity, and thus the bioavailability of the compounds. In fact, some scientific reports indicate that the antitumor activity of bisphosphonate esters is in many cases significantly higher than that of the corresponding acids.^22,23^ For those reasons, bisphosphonate esters are considered attractive candidates for the treatment of both skeletal and non-skeletal tumors.

The potential of bisphosphonates in cancer therapies can be further utilized using a conjugation strategy, which involves chemically binding bisphosphonates to other biologically active compounds. The benefit of using such molecular hybrids may be not only the synergistic and/or additive effects of the two compounds but also the targeted action of the resulting conjugate due to the presence of phosphonic groups, which, when hydrolyzed, could act as a bone-directing carrier. Bisphosphonates have been successfully conjugated with various drugs, antibiotics, and other biologically active compounds.^24,25^ Despite promising research results, none of the described molecular hybrids have been put into clinical practice so far.

An interesting example of a biologically attractive natural parent molecule is betulin (BN). BN is cheap, easily accessible from natural resources and its isolation procedures are well-developed.^26^ BN exhibits a wide spectrum of biological activity, including: anticancer, antibacterial and antiviral.^27–31^ The anticancer activity of BN and its derivatives has been repeatedly confirmed in *in vitro* and *in vivo* studies, including the treatment of melanoma, neuroblastoma or hepatoma.^30,31^ As a natural compound, betulin has a high safety profile, which makes it a very promising agent for medical purposes. The biggest limitation preventing BN from being used as therapeutic is its poor solubility and bioavailability resulting from its highly hydrophobic structure.^26^ However, the presence of easily transformable functional groups (including C-3OH, C-28OH) enables the formation of BN's semi-synthetic derivatives with improved pharmacokinetic properties.

**Fig. 1:**
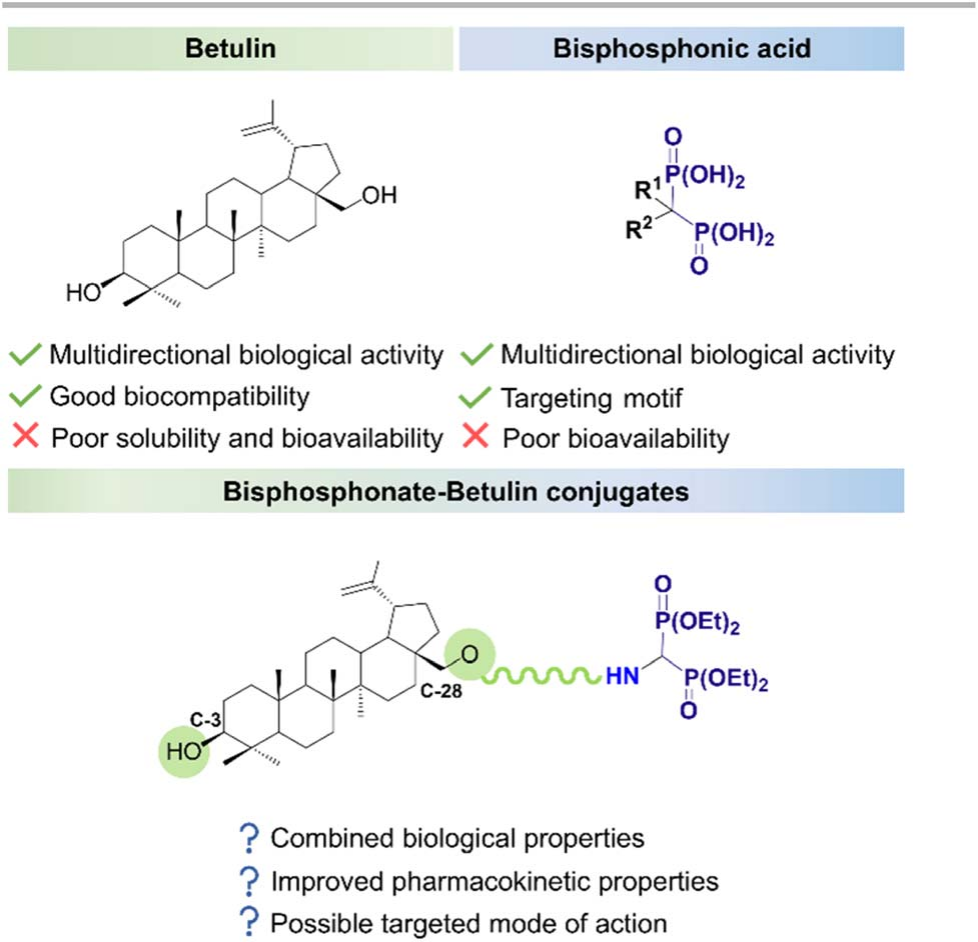
Bisphosphonate–betulin conjugates – potential benefits.

To combine the biological/anticancer properties of bisphosphonates and betulin we have designed and then developed a simple and efficient way to produce molecular hybrids based on a backbone of natural betulin and synthetic tetraethyl α-aminobisphosphonate, combined by an amide bond through a linker (Fig. 1). The carboxyacyl moiety seemed to be a suitable linker, as it allowed further structural modifications of the molecule. Furthermore, numerous literature reports suggest that linking a triterpene backbone with this type of moiety can improve its biological properties, including antibacterial, antifungal, or anti-HIV activity.^29,32^ We synthesized three molecular hybrids, differing in the type of linker used ((CO)CH_2_CR_2_COOH, R = H or Me) and the number of bisphosphonate moieties attached (mono- 4a, 4b, or disubstituted 6 derivatives). Then we evaluated their cytotoxic effect on two non-skeletal and one skeletal cell lines: lung adenocarcinoma (A549), gastric adenocarcinoma (AGS), and osteosarcoma (U-2 OS). Only one conjugate of this type has been described in the literature so far (betulinic acid – tetraethyl 1-aminoethylidene-1,1-bisphosphonate conjugate). Despite the high yield of the product, the authors did not provide any information on its biological activity.^33^

## Results and discussion

### Conjugates synthesis

**Scheme 1:**
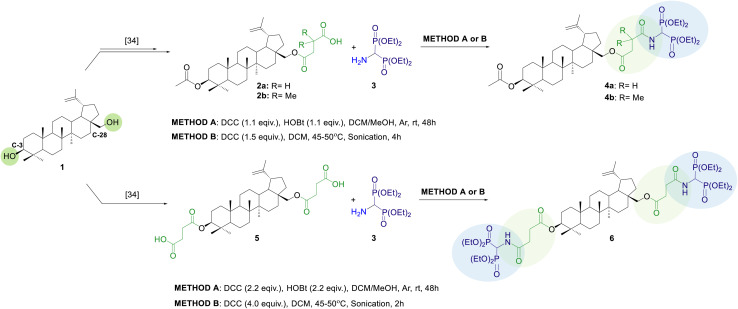
Synthetic route for the preparation of bisphosphonate–betulin conjugates 4a, 4b, 6.

The strategy used in the synthesis of conjugates 4a, 4b, and 6 involved the introduction of the linker into the betulin structure 1 at C-28 (monosubstituted BN analogs 2a, 2b) or C-3 and C-28 positions (disubstituted BN analog 5), followed by coupling the appropriate betulin building block with tetraethyl 1-aminomethylidene-1,1-bisphosphonate 3 (Scheme 1).

BN analogs 2a, 2b and 5 were prepared following published procedures, with some modifications described by us recently.^34^ The monosubstituted derivatives 2a, 2b were obtained in a three-step synthesis, that included (i) acylation of betulin, (ii) selective deprotection of the C-28 position, and (iii) subsequent reaction with succinic anhydride (SA) or 2,2-dimethylsuccinic anhydride (DMSA), while the disubstituted derivative 5 was prepared directly by reacting betulin with succinic anhydride (Scheme 1).

**Scheme 2:**
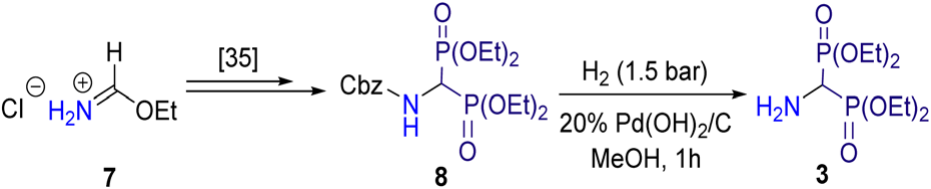
Synthesis of tetraethyl 1-aminomethylidene-1,1-bisphosphonate 3.

The second building block, tetraethyl 1-aminomethylidene-1,1-bisphosphonate 3, was obtained by catalytic hydrogenation of its *N*-Cbz protected analog 8 in a Parr apparatus using 20% palladium hydroxide deposited on activated charcoal in a 99% yield. The *N*-Cbz bisphosphoric derivative 8 was prepared in a four-step synthesis, according to our recently published protocol.^35^ The procedure involved the acylation of the starting ethyl formimidate hydrochloride 7 with benzyl chloroformate, followed by a Michaelis–Becker-like addition of diethyl phosphite to the ethyl *N*-Cbz formimidate, subsequent treatment of the 1-ethoxymethylphosphonate obtained with triphenylphosphonium tetrafluoroborate, and finally Michaelis–Arbuzov-type reaction of the resulting *N*-Cbz protected α-(diethoxyphosphoryl)phosphonium tetrafluoroborate with triethyl phosphite (Scheme 2).

The synthesis of the conjugates began with preliminary experiments in which we screened various reaction conditions for the amide bond formation between aminobisphosphonate 3 and 3-*O*-acetyl-28-*O*′-(3′-carboxypropanoyl)betulin 2a (Table 1), used as a model substrate. The reactions were carried out using *N*,*N*′-dicyclohexylcarbodiimide (DCC) as a coupling agent. The procedure involved mixing the betulin analog with DCC for 2 h to produce the active ester, and then subjecting it to nucleophilic substitution by bisphosphonate 3 (two steps/one-pot reaction, Method A). Optimization of the procedure included selection of the molar ratio of reagents, type of solvent, reaction time, and effect of addition of 1-hydroxybenzotriazole (HOBt) (Table 1). A crucial factor was the selection of an appropriate reaction environment to ensure contact between the reactants. It has been shown that it is most beneficial to carry out the reaction in DCM with the addition of methyl alcohol (10%) (Table 1, entries 1–3). As expected, extending the reaction time generally improved substrate 2a conversion, however, performing the reaction for more than 2 days did not seem particularly beneficial due to a decrease in reaction rate (Table 1, entries 1–3). Increasing the molar excess of bisphosphonate (Table 1, entry 4) and the addition of 1-hydroxybenzotriazole (Table 1, entry 5) turned out to be beneficial, resulting in an increase in substrate conversion of approximately 10% in each case (compared to entry 1). Next, with optimal conditions in hand, the expected products 4a, 4b and 6 were synthesized and isolated by extraction from the solid phase with ethyl acetate and subsequent purification by double column chromatography in 34–77% yields (Table 1, entries 6–8). The structures of all synthesized compounds (4a, 4b and 6) were confirmed by spectroscopic methods (^1^H, ^13^C, ^31^P NMR, FTIR and HRMS).

**Table 1:** Synthesis of bisphosphonate–betulin conjugates 4a, 4b, and 6 – optimization of reaction conditions and yields

Entry	BN analog	Molar ratio of BN analog : 3 : DCC : HOBt	Solvent	Time, h	Temp., °C	Product	Conversion^a^, %	Yield^b^, %
**Method A**								
1	2a	1.0 : 1.5 : 1.5 : —	DCM + MeOH	24/48/144	r.t.	4a	42/54/55	—
2	2a	1.0 : 1.5 : 1.5 : —	AcOEt + MeOH	24/48/144	r.t.	4a	47/51/52	—
3	2a	1.0 : 1.5 : 1.5 : —	MeCN + MeOH	24/48/144	r.t.	4a	41/42/42	—
4	2a	1.0 : 2.0 : 1.5 : —	DCM + MeOH	24	r.t.	4a	56	—
5	2a	1.0 : 1.5 : 1.5 : 1.1	DCM + MeOH	24	r.t.	4a	56	—
6	2a	1.0 : 2.0 : 1.1 : 1.1	DCM + MeOH	48	r.t.	4a	73	61
7	2b	1.0 : 2.0 : 1.1 : 1.1	DCM + MeOH	48	r.t.	4b	79	77
8	5	1.0 : 4.0 : 2.2 : 2.2	DCM + MeOH	48	r.t.	6	—c	34

**Method B**								
9	2a	1.0 : 2.0 : 1.5	DCM	2/4	45–50	4a	94/96	—/91
10	2b	1.0 : 2.0 : 1.5	DCM	2/4	45–50	4b	90/97	—/92
11	5	1.0 : 4.0 : 4.0	DCM	2	45–50	6	—c	69

a Determined by ^1^H NMR spectra analysis.

b Isolated.

c Impossible to determine due to signals overlap.

Knowing that amide bond formation can be facilitated by sonication,^36^ a series of ultrasound-assisted reactions were conducted. This time, only DCC was used for coupling, and the active ester generation step was omitted. All reactants were dissolved in dichloromethane and the reaction was carried out at 45–50 °C under ultrasound irradiation (Method B). The use of ultrasound not only reduced the reaction time from several days to several hours but also improved the overall yield of the product. For monosubstituted analogs 4a, 4b, excellent substrate conversion (96–97% based on NMR analysis) was observed after 4 hours. In the case of derivative 6, despite of incomplete substrate conversion, the optimal reaction time was found to be 2 hours, as a further reaction led to the formation of some side products. Finally, after purifying the products as in Method A, they were obtained with 69–92% yields.

Both proposed methods have their advantages and disadvantages. The ultrasound-assisted reaction procedure (Method B) is simpler than the step-by-step protocol proposed in Method A. Additionally, sonication allowed us to shorten the total reaction time from 48 hours to 2 hours for conjugate 6 and 4 hours for conjugates 4a, 4b. The products synthesized according to Method B were obtained with higher yields (69–92%) compared to conjugates obtained by Method A (39–77%). In Method B the greater excess of DCC was used than in Method A; however, the need for a coupling additive (HOBt) was eliminated. Although the purification process of the final products in both methods included extraction and double column chromatography, the isolation of pure conjugates obtained by Method B was facilitated by the lower content of unreacted substrates and the absence of HOBt in the reaction mixture. The advantage of Method A, on the other hand, is that the reaction is carried out at room temperature and does not require unconventional methods such as sonication.

### Biological studies

The cytotoxic activity of bisphosphonate conjugates with betulin (4a, 4b, and 6) was tested in 3 different human cancer cell lines: U-2 OS (RRID: CVCL_0042, osteosarcoma, American Type Culture Collection [ATCC], Manassas, VA, USA), A549 (RRID: CVCL_0023, lung adenocarcinoma, ATCC), AGS (RRID: CVCL_0139, gastric adenocarcinoma, ATCC) and 1 normal cell line: BJ1-hTERT (RRID: CVCL_6573, immortalized human skin fibroblasts, ATCC). The choice of cell lines was based on the reported effects of bisphosphonates and betulin. Bisphosphonates, as bone resorption inhibitors, are known to prevent skeletal-related events, reduce pain, and increase survival in patients with bone metastatic lung cancer, breast cancer, and prostate cancer.^37,38^ In addition, numerous studies have demonstrated their direct effects on several cancer cell lines *in vitro*, including osteosarcoma cell lines^39,40^ and non-small cell lung cancer cell lines.^41,42^ The effect of bisphosphonates on lung metastases to bones is also being studied; however, the results reported in the literature so far are contradictory.^43^ Betulin, as a compound with multidirectional biological activity, has also demonstrated its activity against various types of cancers, including lung cancer (A549, H1264, and Calu-6 cell lines)^44^ and gastrointestinal cancers such as colorectal cancer (i.a. SW707, HT29, HCT116 cell lines)^45,46^ or gastric cancer (SGC7901 cell line).^47^

In order to determine the IC50 values for each conjugate in each of the cell lines examined, the MTS test (Promega) was used. The cytotoxic effect of the conjugates tested was then compared with the effect of betulin itself. The stock solutions of betulin and bisphosphonate conjugates with betulin (4a, 4b, and 6) were prepared in DMSO. At this point, we observed that all tested conjugates had better solubility in DMSO (>30 mM) compared to betulin (5 mM). Then, the stock solutions were diluted in culture media to concentrations that were experimentally determined for each substance in each of the cell lines, in which the cytotoxic effects were evaluated. The concentration ranges are summarized in Table 2.

**Table 2:** Concentration ranges of conjugates 4a, 4b, 6, and betulin (BN) in culture media in μM

Compound	Cell line			
	U-2 OS	A549	AGS	BJ1-hTERT
4a	0–30	0–50	0–35	0–35
4b	0–35	0–60	0–50	0–50
6	0–14	0–14	0–12	0–20
BN	0–20	0–18	0–18	0–40

**Fig. 2:**
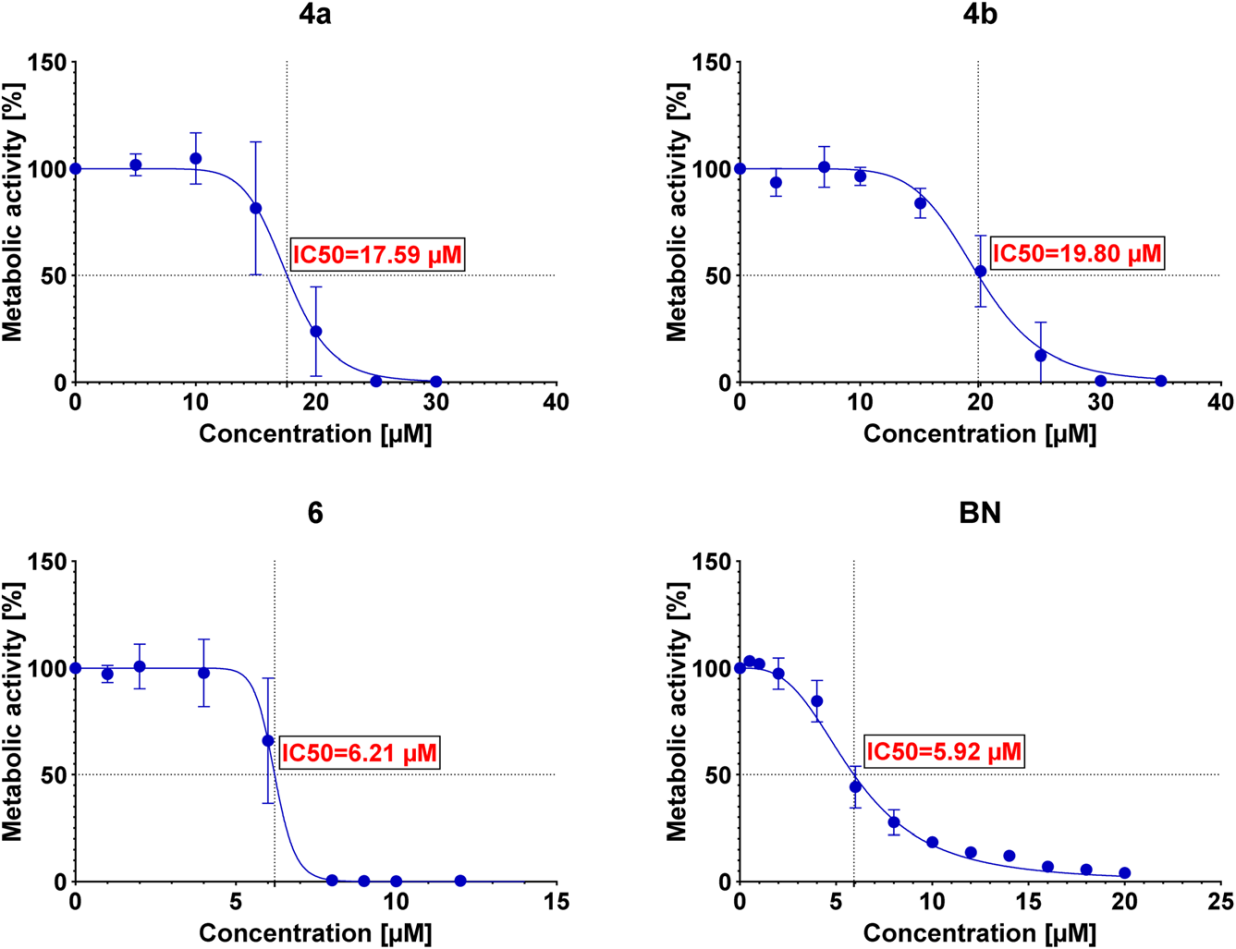
Graphs showing the dependence of the metabolic activity of U-2 OS cells on the concentration of conjugates 4a, 4b, and 6 and betulin (BN).

**Fig. 3:**
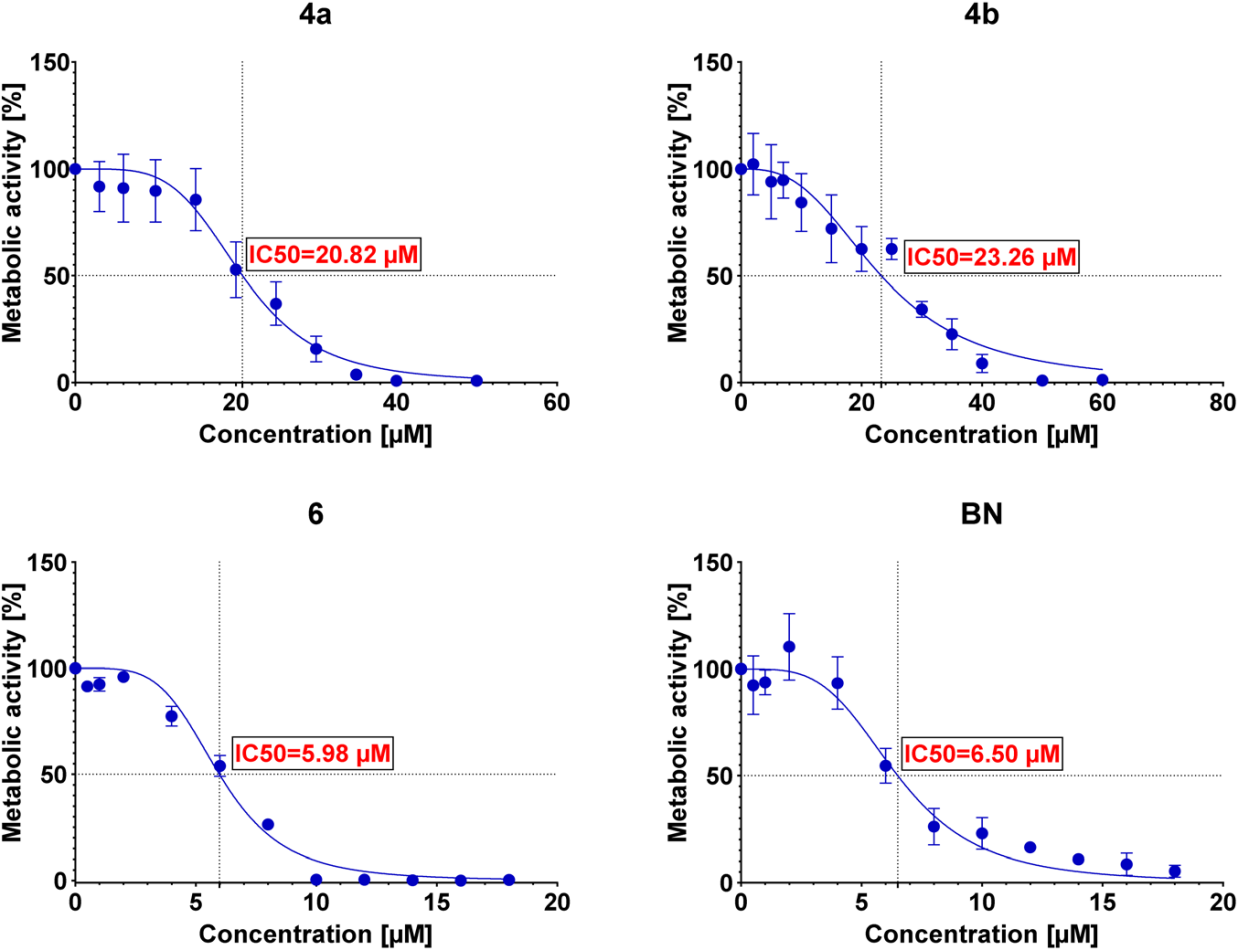
Graphs showing the dependence of the metabolic activity of A549 cells on the concentration of conjugates 4a, 4b, and 6 and betulin (BN).

**Fig. 4:**
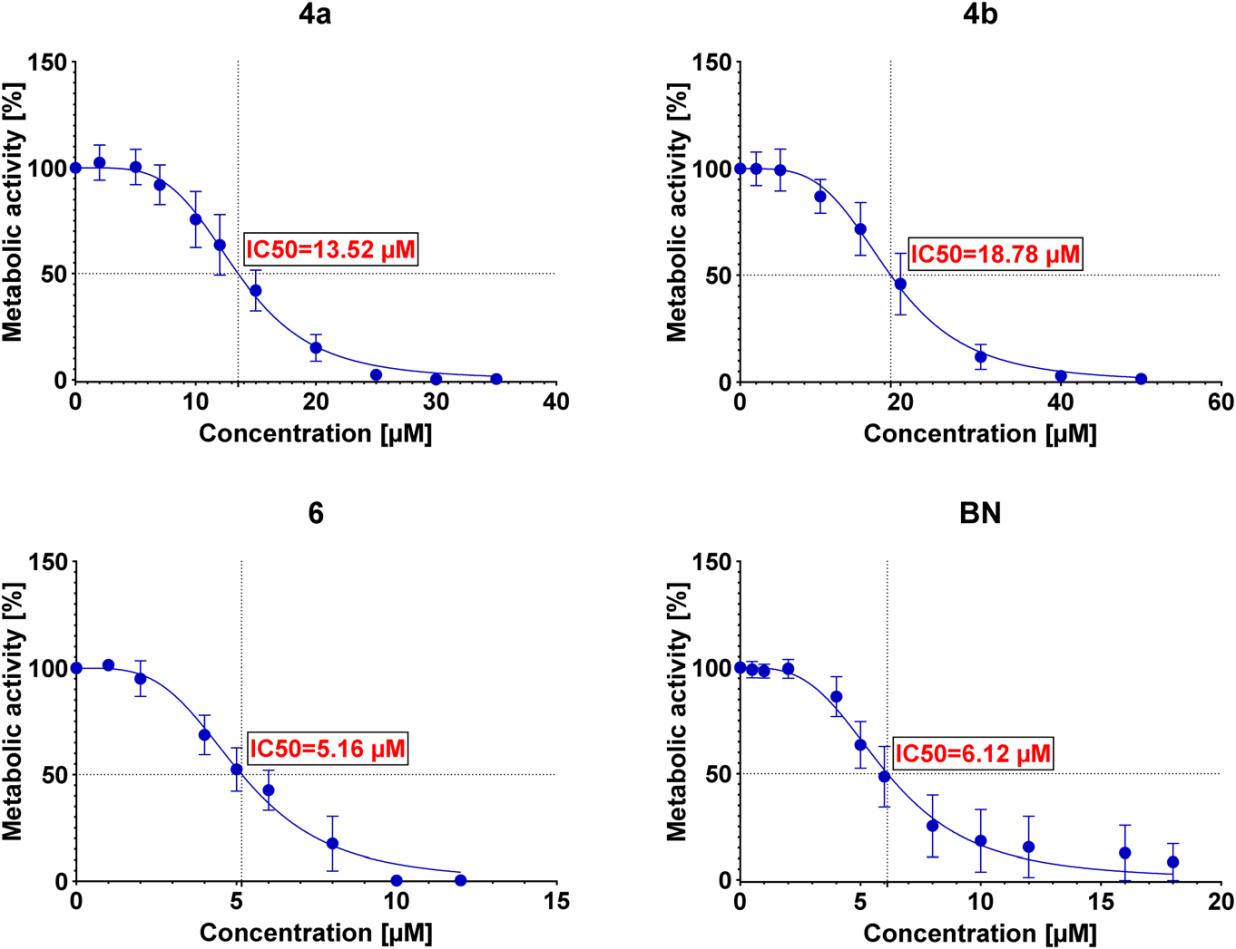
Graphs showing the dependence of the metabolic activity of AGS cells on the concentration of conjugates 4a, 4b, and 6 and betulin (BN).

**Fig. 5:**
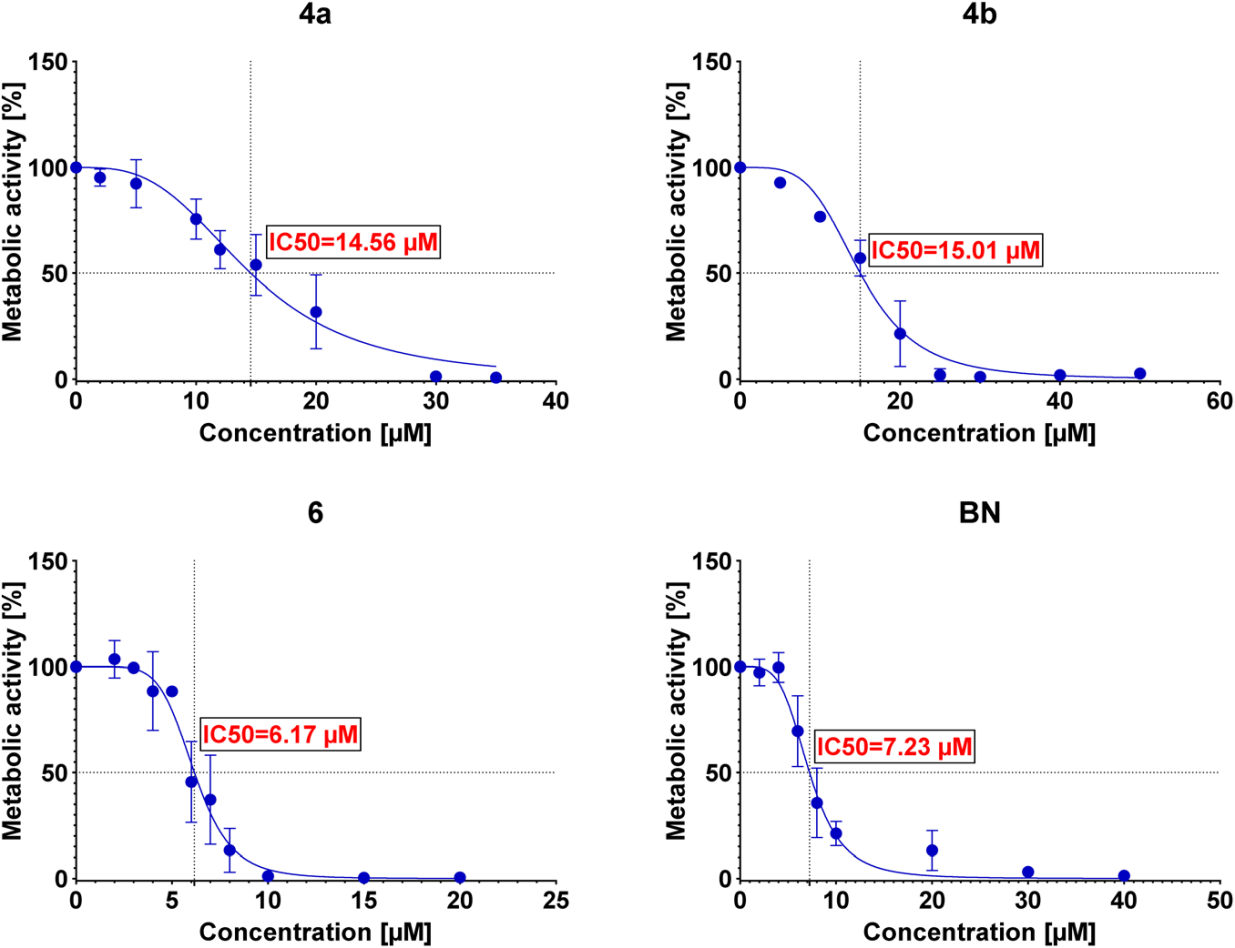
Graphs showing the dependence of the metabolic activity of BJ1-hTERT cells on the concentration of conjugates 4a, 4b, and 6 and betulin (BN).

The IC50 values were then determined for each conjugate tested in each of the examined cell lines. The determined IC50 values are summarized in Table 3, and the curves are presented in Fig. 2–5.

**Table 3:** Comparison of *in vitro* determined IC50 values for conjugates 4a, 4b, 6 and betulin (BN) for U-2 OS, A549, AGS and BJ1-hTERT cell lines

Compound	*In vitro* cytotoxic activity^a^, IC50, μM			
	U-2 OS	A549	AGS	BJ1-hTERT
4a	17.59	20.82	13.52	14.56
4b	19.80	23.26	18.78	15.01
6	6.21	5.98	5.16	6.17
BN	5.92	6.50	6.12	7.23

a Cytotoxicity was assessed using the MTS test after 48 h of incubation. IC50 values are expressed in μM.

Preliminary cytotoxicity studies of the obtained compounds showed that the highest activity exhibits the conjugate 6. The IC50 values (between 5.16 μM and 6.21 μM) in various cell lines indicate that the cytotoxicity of this conjugate is comparable (for A549 and AGS even slightly better) to the cytotoxicity of betulin itself (between 5.92 μM and 7.23 μM). However, its use can be limited by the lack of selectivity toward cancer cell lines – IC50 values are similar not only among cancer cell lines but also towards normal cells (as in the case of betulin). Conjugates 4a and 4b show similar cytotoxicity (to each other); however, in these cases, the conjugation of betulin with bisphosphonate reduces its activity. Moreover, like conjugate 6, these conjugates do not show selectivity towards cancer cells. The greatest influence on the biological activity of the conjugates obtained has the number of bisphosphonate moieties attached and/or the site of their attachment. Conjugate 6 with two bisphosphonate units, at C-3 and C-28 positions, shows about 3-fold higher cytotoxicity compared to monosubstituted conjugates 4a and 4b. It is worth noting that the type of linker used has a small but noticeable effect on the cytotoxic activity of these conjugates (compare IC50 values of 4a and 4b). Although conjugation did not significantly improve the cytotoxic activity of betulin, all tested conjugates were characterized by much better solubility in DMSO (>20 mg mL^−1^) compared to betulin itself (5 mg mL^−1^) which may positively affect their bioavailability. Additionally, the information on structure–activity correlation can facilitate the design of new BP–BN conjugates in the future.

## Conclusions

In summary, in this work we have proposed an efficient method for the synthesis of three new betulin–bisphosphonate conjugates. We have shown that supporting the amide bond formation with ultrasound eliminates the need for a coupling additive, shortens the reaction time (from 48 to 2–4 h) and improves the yields of the products. Among the tested compounds, the best prognosis was shown by the conjugate with two bisphosphonate units, which was characterized by higher cytotoxicity and better solubility than betulin. However, its use can be limited by a lack of selectivity toward cancer cells. The results of biological research indicate that the idea of conjugation of bisphosphonate with betulin may, with some additional modifications, prove to be valid for improving the biological activity and solubility of betulin and the proposed method of conjugation may prove useful in the synthesis of other molecular hybrids linked by an amide bond.

## Data availability

The data supporting this article have been included as a part of the ESI.† ESI includes synthetic procedures, compound characterization, ^1^H, ^13^C, and ^31^P NMR spectra, HRMS, IR, colorimetric cell viability assay protocol (PDF).

## Author contributions

Conceptualization, M. G. and A. K.; formal analysis, D. K., M. G., M. K., B. Ł.-S.; investigation, D. K., M. K., B. Ł.-S.; methodology, M. G., A. K., M. K., J. A.; supervision, A. K., M. G., J. A., T. C.; writing – original draft, D. K.; writing – review & editing, D. K., A. K., M. K., J. A. All authors have read and agreed to the published version of the manuscript. The manuscript was written through contributions of all authors. All authors have given approval to the final version of the manuscript.

## Conflicts of interest

There are no conflicts to declare.

## Supplementary Material

RA-015-D4RA07782B-s001
