# Supplementary material for: Ultrasound-assisted synthesis of new bisphosphonate–betulin conjugates and preliminary evaluation of their cytotoxic activity
Source: RSC Adv. 2025 Feb 6;15(6):4086–94. doi: 10.1039/d4ra07782b (PMC11800102; doi:10.1039/d4ra07782b)
Supplement: RA-015-D4RA07782B-s001 [file RA-015-D4RA07782B-s001.pdf]

Supplementary Information (SI) for RSC Advances.
This journal is © The Royal Society of Chemistry 2025

Supporting Information

Ultrasound-assisted synthesis of new bisphosphonate-betulin conjugates and preliminary evaluation of their cytotoxic activity

Dominika Kozicka,[*ab]‡ Małgorzata Krześniak,[c]‡ Mirosława Grymel,[ab] Jakub Adamek,[ab] Barbara Łasut-Szyszka,[c] Tomasz Cichoń[c] and Anna Kuźnik[*ab]

[a]Department of Organic Chemistry, Bioorganic Chemistry and Biotechnology, Silesian University of Technology, B. Krzywoustego 4, 44-100 Gliwice, Poland

[b]Biotechnology Center, Silesian University of Technology, B. Krzywoustego 8, 44-100 Gliwice, Poland

[c]Center for Translational Research and Molecular Biology of Cancer, Maria Skłodowska-Curie National Research Institute of Oncology, Gliwice Branch, Wybrzeże Armii Krajowej 15, 44-102 Gliwice, Poland

* Correspondence: dominika.kozicka@polsl.pl, anna.kuznik@polsl.pl

Table of Contents

1. Details of chemical synthesis…S2
   a. General information…S2
   b. Experimental procedures…S2
2. Characterization data of compounds **3**, **4a**, **4b**, **6**…S4
3. Details of biological assay…S6
   a. Cell culture and treatment…S6
   b. Colorimetric cell viability assay…S6
   c. Statistical analysis…S7
4. NMR spectra of compounds **4a**, **4b**, **6**…S8
5. References…S12

1. Details of chemical synthesis

a. General information

Ultrasound assisted reactions were carried out using an Elmasonic S 10 H ultrasound device with a frequency of 37 kHz and a nominal power of 90 W. Reactions were monitored by TLC analysis using commercially available Merck TLC silica gel 60 F254 plates. The TLC plates were visualized under UV light (λ = 254 nm) or by charring after spraying with a 10% solution of sulfuric acid in ethanol. The purification of the crude compounds was carried out by column chromatography using silica gel (Merck, 0.040–0.063 mm). No unexpected or unusually high safety hazards were encountered. Solvents (ACS grade) were stored over molecular sieves before use. All other commercially available reagents were purchased from commercial suppliers and used without further purification. 3-*O*-acetyl-28-*O*'-(3'-carboxypropanoyl)betulin **2a**, 3-*O*-acetyl-28-*O*'-(3'-carboxy-3,3-dimethylpropanoyl)betulin **2b**, 3,28-*O,O'*-bis(3-carboxypropanoyl)betulin **5** and tetraethyl 1-(*N*-benzyloxycarbonylamino)methylene-1,1-bisphosphonate **8** were prepared according to our previously described procedures.[1,2] The ^{1}H and ^{13}C NMR spectra were recorded on a Varian spectrometer at operating frequencies of 600 and 100 MHz, respectively. Chemical shifts are reported relative to TMS (tetramethylsilane) used as an internal shift standard (^{1}H NMR, zero ppm) or residual chloroform (^{13}C NMR, 77.16 ppm). The 31P-NMR spectra were recorded at an operating frequency of 161.9 MHz, with respect to H_3PO_4 at zero ppm. All chemical shifts (δ) are reported in ppm and coupling constants (*J*) in Hz. The following abbreviations were used to explain the observed multiplicities: s-singlet; d-doublet; dd-double doublet; t-triplet; m-multiplet; br-broad. Infrared (IR) spectra were measured on a Nicolet 6700 Fourier transform (FT)-IR spectrophotometer (Attenuated Total Reflectance - ATR method). High-resolution mass spectrometry (HR-MS) analyzes were performed using a Waters Xevo G2 Q-TOF mass spectrometer equipped with an ESI source operating in positive ion mode. The accurate mass and composition of the molecular ion adducts were calculated using the MassLynx 4.1 software incorporated within the instrument.

b. Experimental procedures

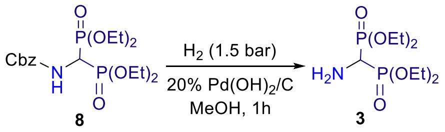

The catalytic hydrogenation of 1-(*N*-benzyloxycarbonylamino)methylene-1,1-bisphosphonate 8

To a solution of 1-(*N*-benzyloxycarbonylamino)methylene-1,1-bisphosphonate **8** (1.29 g, 2.95 mmol) in methanol (20 mL) 20% $Pd(OH)_2/C$ (295 mg) was added. The reaction was carried out in the Paar hydrogenation apparatus at 1.5 bar hydrogen pressure for 1 h at room temperature. The reaction mixture was filtered through a plug of celite and condensed to obtain pure product **3** as pale yellow oil in 99% yield.

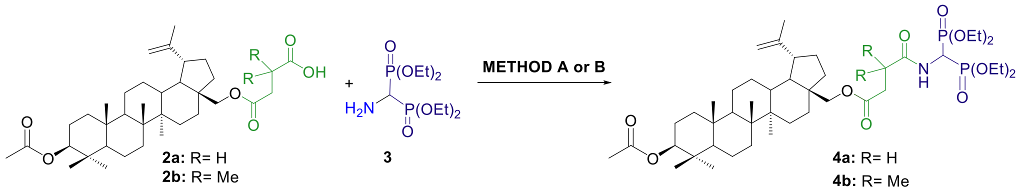

METHOD A: DCC (1.1 eqiv.), HOBt (1.1 eqiv.), DCM/MeOH, Ar, rt, 48h
METHOD B: DCC (1.5 equiv.), DCM, 45-50°C, Sonication, 4h

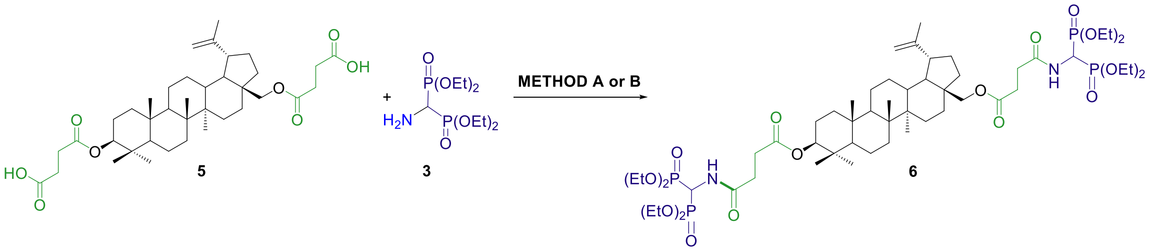

METHOD A: DCC (2.2 eqiv.), HOBt (1.1 eqiv.), DCM/MeOH, Ar, rt, 48h
METHOD B: DCC (4.0 equiv.), DCM, 45-50°C, Sonication, 2h

Procedure for the synthesis of 4a,b and 6 (METHOD A)

To a round-bottomed flask appropriate betulin derivative **2a** or **2b** (0.10 mmol, 1.0 equiv.), 1-hydroxybenzotriazole (14.9 mg, 0.11 mmol, 1.1 equiv.) and *N,N*-dicyclohexylcarbodiimide (22.7 mg, 0.11 mmol, 1.1 equiv.) were added. The flask was flushed with argon and then dichloromethane (4.5 mL) was added. The mixture was stirred at 0-5°C for 15 minutes and then at room temperature for 2 hours. Next, solution of tetraethyl 1-amino-1,1-methylidenebisphosphonate **3** (60.6 mg, 0.2 mmol, 2.0 equiv.) in dichloromethane (2.4 mL) was added dropwise at 0°C, followed by the addition of methanol (0.25 mL). The reaction was continued at room temperature for a total of 48 hours. After the reaction completed, the solvent was evaporated under reduced pressure and the residue was extracted with ethyl acetate (3x3 mL) to separate the dicyclourea. The extracts were combined, organic solvent was removed under reduced pressure and the obtained residue was further purified by double column chromatography (AcOEt/MeOH, gradient 20:1 to 5:1), providing pure compounds **2a** and **2b** in yields of 61% and 77%, respectively.

The synthesis, isolation, and purification of compound **6** were performed in analogous manner, but starting from the betulin derivative **5** (64.3 mg, 0.10 mmol, 1.0 equiv.), and using a larger excess of 1-hydroxybenzotriazole (29.8 mg, 0.22 mmol, 2.2 equiv.), *N,N*-dicyclohexylcarbodiimide (45.4 mg, 0.22 mmol, 2.2 equiv.) and 1-amino-1,1-methylidenebisphosphonate (121.2 mg, 0.4 mmol, 4.0 equiv.).

Procedure for the synthesis of 4a,b and 6 (METHOD B)

To a glass vial sealed with a screw-cap tetraethyl 1-amino-1,1-methylidenebisphosphonate **3** (60.6 mg, 0.2 mmol, 2.0 equiv.), appropriate betulin derivative **2a** or **2b** (0.10 mmol, 1.0 equiv.), *N,N*-dicyclohexylcarbodiimide (30.9 mg, 0.15 mmol, 1.5 equiv.) and dichloromethane (5 mL) were added. The reaction mixture was purged with argon and reaction was carried out at 45-50°C for 4 hours under

ultrasound irradiation. Expected products **4a** and **4b** were isolated and purified as in method A and obtained in 91% and 92% yields respectively.

Conjugate **6** was synthesized in analogous manner, but starting from betulin derrivative **5** (64.3 mg, 0.10 mmol, 1.0 equiv.), and using larger excess of *N,N*-dicyclohexylcarbodiimide (82.5 mg, 0.4 mmol, 4.0 equiv.) and 1-amino-1,1-methylidenebisphosphonate (121.2 mg, 0.4 mmol, 4.0 equiv.). The reaction was carried out for 2 hours. Expected products **6** was isolated and purified as in method A and obtained in 69% yield.

2. Characterization data of compounds 3, 4a, 4b, 6

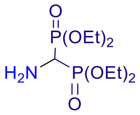

Tetraethyl aminomethylbis(phosphonate) (3). Pale yellow oil; 99% yield. **^{1}H NMR** (400 MHz, $CDCl_3$): δ_H 1.36 (t, J = 7.1 Hz, 12H, CH_3), 3.42 (t, J = 20.6 Hz, 1H, $NH_2C\underline{H}$), 4.21-4.25 (m, 8H, CH_2) ppm; **^{13}C NMR (100 MHz, $CDCl_3$):** δ_C 16.6 (d, J = 2.9 Hz), 16.6 (d, J = 3.0 Hz), 47.9 (t, J = 144.6 Hz), 63.3 (d, J = 3.3 Hz), 63.3 (d, J = 2.8 Hz), 63.3 (d, J = 2.5 Hz), 63.3 (d, J = 3.2 Hz) ppm; **^{31}P NMR** (162 MHz, $CDCl_3$): δ_P 20.2 ppm. NMR data in agreement with literature data.[3]

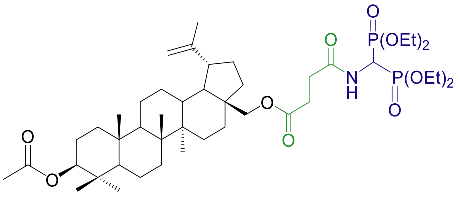

3-OAc-28-SANHCH(PO(OEt)$_2$)$_2$ BN (4a). Colorless resin; R_f = 0,5 (AcOEt/MeOH 5:1); 61% yield (method A), 91% yield (method B). **^{1}H NMR** (600 MHz, $CDCl_3$): δ_H 0.83, 0.84, 0.97, 1.02 (4 x s, 15H, H-23–H-27), 1.336 (t, J = 7.2 Hz, 6H 2 x $OCH_2C\underline{H}_3$), 1.339 (t, J = 7.2 Hz, 6H, 2 x $OCH_2C\underline{H}_3$), 1.68 (s, 3H, H-30), 0.77–1.92 (m, 24H, CH, CH_2), 2.04 (s, 3H, CH_3CO), 2.42 (td, J_1 = 5.7 Hz, J_2 = 11.0 Hz, 1H, H-19), 2.58 (t, J = 7.0 Hz, 2H, $O(CO)CH_2CH_2$), 2.69 (t, J = 7.2 Hz, 1H, 2H, $O(CO)CH_2CH_2$), 3.87 (d, J = 11.1 Hz, 1H, H-28b), 4.15–4.24 (m, 8H, 4 x $OC\underline{H}_2CH_3$), 4.28 (d, J = 11.1 Hz, 1H, H-28a), 4.46 (dd, J_1 = 5.3 Hz, J_2 = 11.1 Hz, 1 H, H-3), 4.58-4.59 (m, 1H, H-29b), 4.68 (d, J = 2.6, 1H, H-29a), 5.01 (td, J_1 = 10.1 Hz, J_2 = 21.7 Hz, 1H, $NHC\underline{H}$), 6.19 (br d, J = 9.8 Hz, 1H, NH) ppm; **^{13}C NMR (100 MHz, $CDCl_3$):** δ_C 14.9, 16.1, 16.3, 16.4 (d, J = 3.1 Hz), 16.5 (d, J = 3.1 Hz), 16.5 (d, J = 3.0 Hz), 16.6 (d, J = 2.9 Hz), 16.6, 18.3, 19.2, 20.9, 21.4, 23.8, 25.3, 27.2, 28.1, 29.5, 29.7, 29.9, 30.9, 34.2, 34.7, 37.2, 37.7, 37.9, 38.5, 41.0, 42.8, 43.8 (t, J = 146.1 Hz), 46.6, 47.9, 48.9, 50.4, 55.5, 63.2, 63.67 (d, J = 3.2 Hz), 63.7 (d, J = 3.2 Hz), 63.8 (d, J = 3.1 Hz), 63.8 (d, J = 3.1 Hz), 81.0, 110.0, 150.2, 170.6 (t, J = 4.1 Hz), 171.1, 173.0 ppm; **^{31}P NMR** (162 MHz, $CDCl_3$): δ_P 16.2 ppm; **IR (ATR)** ν:

2923, 1732, 1247, 1035 cm^{-1}; **HRMS (ESI$^+$)**: calcd. for $C_{45}H_{78}N\ O_{11}P_2$ ($[M+H]^+$): *m/z* 870.5050; found.: *m/z* 870.5054.

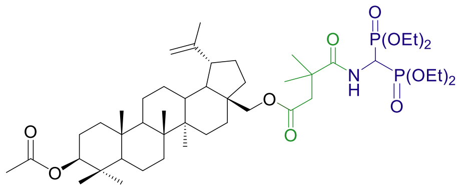

3-OAc-28-DMSANHCH(PO(OEt)$_2$)$_2$ BN (4b). Colorless resin; R_f = 0,55 (AcOEt/MeOH 5:1); 77% yield (method A), 92% yield (method B). **^{1}H NMR** (600 MHz, $CDCl_3$): δ_H 0.83, 0.84, 0.96, 1.01 (4 x s, 15H, H-23–H-27), 1.32 (br s, 6H, 2 x $C(CH_3)_2$), 1.34, 1.35 (2 x t, *J* = 7.2 Hz, 12H, 4 x $OC\underline{H}_2CH_3$), 1.68 (s, 3H, H-30), 0.78–1.94 (m, 24H, CH, CH_2), 2.04 (s, 3H, CH_3CO), 2.41 (td, J_1 = 5.7 Hz, J_2 = 10.9 Hz, 1H, H-19), 2.63 (s, 2H, $O(CO)CH_2$), 3.83 (d, *J* = 11.1 Hz, 1H, H-28b), 4.17–4.26 (m, 9H, H-28b i 4 x $OCH_2C\underline{H}_3$), 4.47 (dd, J_1 = 5.4 Hz, J_2 = 10.9 Hz, 1H, H-3), 4.58-4.59 (m, 1H, H-29b), 4.68 (d, *J* = 2.3 Hz, 1H, H-29a), 5.05 (td, J_1 = 9.9 Hz, J_2 = 21.6 Hz, 1H, $NHC\underline{H}$), 6.53 (br d, *J* = 9.9 Hz, 1H, NH) ppm; **^{13}C NMR** (100 MHz, $CDCl_3$): δ_C 14.8, 16.1, 16.3, 16.4 (d, *J* = 3.1 Hz), 16.5 (d, *J* = 3.2 Hz), 16.5 (d, *J* = 3.0 Hz), 16.6 (d, *J* = 2.9 Hz), 16.6, 18.3, 19.2, 20.9, 21.4, 23.8, 25.3, 25.6, 25.6, 27.1, 28.1, 29.7, 29.9, 34.2, 34.7, 37.2, 37.7, 37.9, 38.5, 40.9, 41.0, 42.8, 43.7 (t, *J* = 145.6 Hz), 43.7, 46.5, 47.9, 48.9, 50.4, 55.5, 62.7, 63.6 (d, *J* = 3.2 Hz), 63.6 (d, *J* = 3.1 Hz), 63.7 (d, *J* = 3.1 Hz), 63.7 (d, *J* = 3.1 Hz), 81.0, 110.0, 150.2, 171.1, 171.8, 175.9 (t, *J* = 3.9 Hz) ppm; **^{31}P NMR** (162 MHz, $CDCl_3$): δ_P 16.5 ppm; **IR (ATR)** ν: 2941, 1733, 1245, 1028 cm^{-1}; **HRMS (ESI$^+$)**: calcd. for $C_{47}H_{82}NO_{11}P_2$ ($[M+H]^+$): *m/z* 898.5363; found: *m/z* 898.5369.

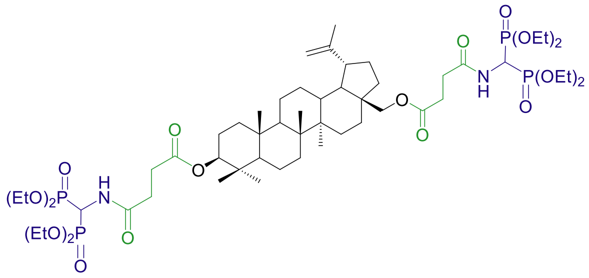

3,28-bis(SANHCH(PO(OEt)$_2$)$_2$) BN (6). Colorless resin; R_f = 0,35 (AcOEt/MeOH 5:1); 34% yield (method A), 69% yield (method B). **^{1}H NMR** (600 MHz, $CDCl_3$): δ_H 0.83, 0.84, 0.97, 1.02 (4 x s, 15H, H-23–H-27), 1.32-1.35 (m, 24H, 8 x $OCH_2C\underline{H}_3$), 1.68 (s, 3H, H-30), 0.76–1.98 (m, 24H, CH, CH_2), 2.42 (td, J_1 = 5.6 Hz, J_2 = 11.2 Hz, 1H, H-19), 2.58-2.70 (m, 8H, 2 x $O(CO)CH_2CH_2$), 3.86 (d, *J* = 11.1 Hz, 1H, H-28b), 4.15–4.22 (m, 16H, 8 x $OC\underline{H}_2CH_3$), 4.28 (d, *J* = 11.1 Hz, 1H, H-28a), 4.47 (dd, J_1 = 6.8 Hz, J_2 = 9.5 Hz, 1H, H-3), 4.58-4.59 (m, 1H, H-29b), 4.68 (d, *J* = 2.5 Hz, 1H, H-29a), 5.03 (td, J_1 = 10.1 Hz, J_2 = 21.7 Hz, 2 x $NHC\underline{H}$), 6.51 (br d, *J* = 10.1 Hz, 2H, 2 x NH) ppm; **^{13}C NMR** (100 MHz, $CDCl_3$): δ_C 14.8, 16.1, 16.2, 16.4–16.5 (m, 8 x $OCH_2\underline{C}H_3$), 16.6, 18.2, 19.2, 20.9, 23.8, 25.3, 27.1, 28.1, 29.4, 29.7, 29.77, 29.83, 30.8, 30.9, 34.2, 34.6, 37.2, 37.7, 37.9, 38.5, 41.0, 42.8, 43.7 (t, *J* = 146.2 Hz),

43.7 (t, *J* = 146.2 Hz), 46.5, 47.8, 48.9, 50.4, 55.5, 63.1, 63.6–63.8 (m, 8 x O$\underline{C}H_2CH_3$), 81. 5, 110.0, 150.2, 170.7 (t, *J* = 7.9 Hz), 170.8 (t, *J* = 8.0 Hz), 172.3, 172.9 ppm; **^{31}P NMR** (162 MHz, $CDCl_3$): δ_P 16.29, 16.28 ppm; **IR (ATR)** ν: 2984, 1737, 1236, 1044 cm^{-1}; **HRMS (ESI$^+$)**: calcd. for $C_{56}H_{101}NO_{18}P_4$ ($[M+H]^+$): *m/z* 1213.6000; found: *m/z* 1213.6082.

3. Details of biological assay

a. Cell culture and treatment

A549 cells (RRID: CVCL_0023, lung adenocarcinoma, American Type Culture Collection [ATCC], Manassas, VA, USA), and U-2 OS cells (RRID: CVCL_0042, osteosarcoma, ATCC) cells were cultured in low glucose (1 g/L) DMEM supplemented with 10% fetal bovine serum (FBS; cat. no. 10270106, Invitrogen, Carlsbad, CA, USA). AGS cells (RRID: CVCL_0139, gastric adenocarcinoma, ATCC) were cultured in McCoy′s 5A supplemented with 10% fetal bovine serum. BJ1-hTERT (RRID: CVCL_6573, immortalized human skin fibroblasts, ATCC) were cultured in a medium with the following composition: 2/3 DMEM with high glucose content (4.5 g/L) and 1/3 Medium 199 (cat.no. M4530, Sigma-Aldrich, St. Louis, MO, USA) supplemented with 10% fetal bovine serum and 0.01 mg/ml hygromycin B-Losung (cat. no. CP12.2, Roth, Karlsruhe, Germany). All cells were cultured at 37°C/5% CO_2 and all media were supplemented with 1% penicilin/streptomycin solution. All culture media and other culture reagents were from Biowest (Biowest, Nuaillé, France) and consumables were from Sarstedt (Sarstedt, Nümbrecht, Germany).

The stock solutions of betulin and bisphosphonate conjugates with betulin (**4a**, **4b** and **6**) were prepared in DMSO. Stock solutions were diluted in culture media to concentrations that were experimentally determined for each tested cell line, and their ranges are presented in **Table 2** (manuscript). Control cells were mock-treated with medium containing DMSO.

b. Colorimetric cell viability assay

Cells were seeded on 96-well plates in the following numbers: U-2 OS – 6 500 cells per well, A549 and AGS – 5 000 cells per well, BJ1-hTERT – 4 000 cells per well. 24 hours after seeding, the cells were treated for 48 hours with various concentrations of conjugates, that were experimentally determined within the ranges shown in **Table 2** (manuscript).

The cytotoxicity of the tested compounds was determined by measuring the metabolic activity of cells. Cell viability was determined using CellTiter 96® AQueous One Solution Cell Proliferation Assay (MTS) (cat. no. G3581, Promega, Madison, WI, USA) kit according to manufacturer’s protocol. The absorbance was read at 490 nm using the BioTek Synergy 2 microplate reader. Data were normalized to control (no conjugates or betulin treatment) – for each compound, the value of 0 μM refers to the metabolic activity of cells treated with DMSO (in which the conjugates and betulin were

dissolved) in an amount corresponding to the highest concentration for each substance used in respective cell line. Each determination was performed in 3 technical repeats and at least 3 biological repeats.

c. Statistical analysis

GraphPad Prism 10.2.2 software (397) (GraphPad Software, Boston, Massachusetts USA, www.graphpad.com) was used to calculate the IC50 and plot the curves. IC50 values were calculated using the method "Inhibitor vs. normalized response - Variable slope".

4. NMR spectra of compounds 4a, 4b, 6

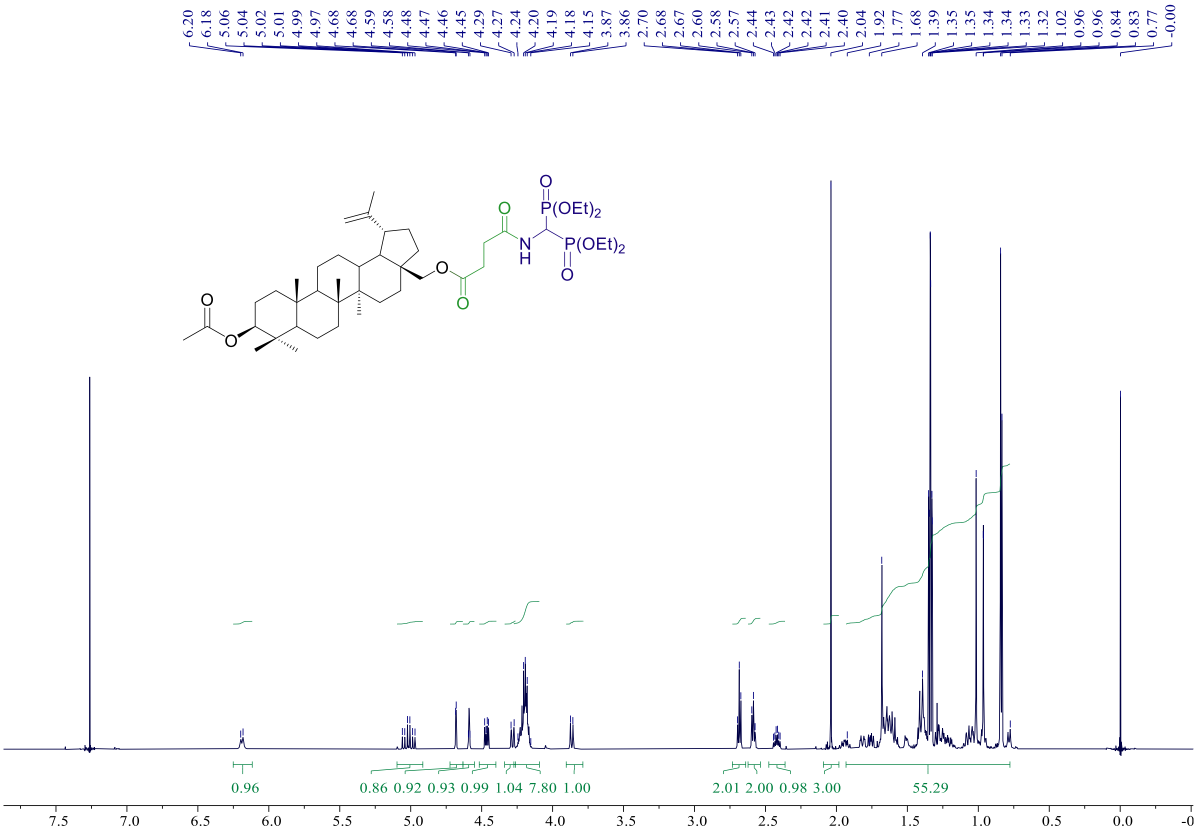

^{1}H NMR spectrum of 3-OAc-28-SANHCH(PO(OEt)$_2$)$_2$ BN (**4a**); 600 MHz/$CDCl_3$/TMS; δ (ppm).

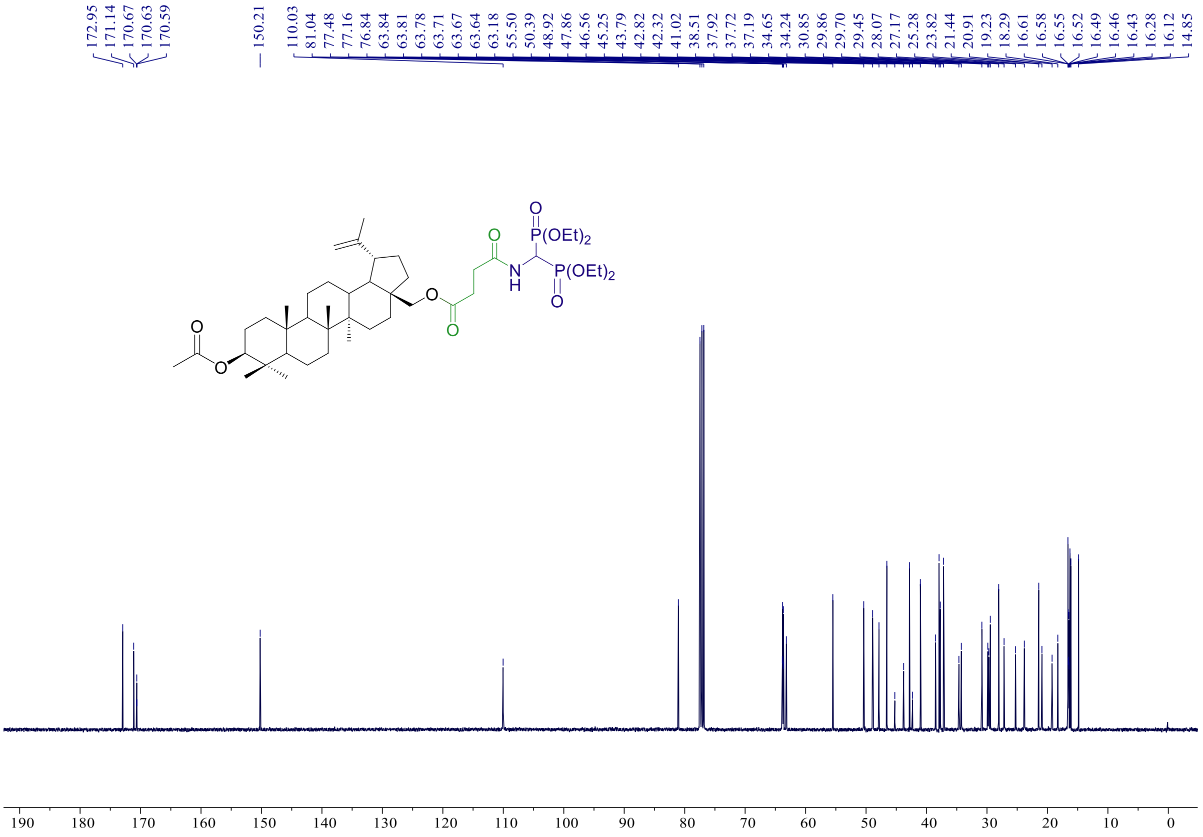

^{13}C NMR spectrum of 3-OAc-28-SANHCH(PO(OEt)$_2$)$_2$ BN (**4a**); 100 MHz/$CDCl_3$/TMS; δ (ppm).

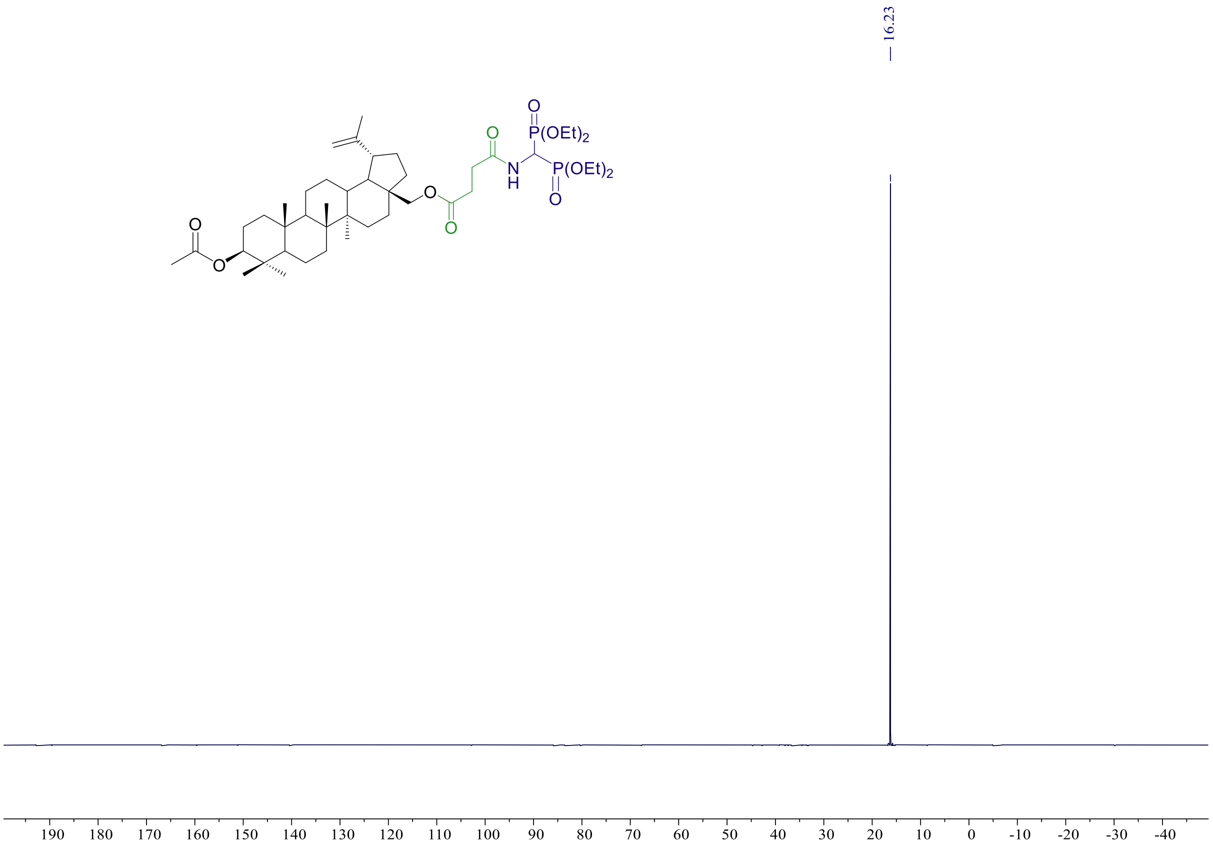

^{31}P NMR spectrum of 3-OAc-28-SANHCH(PO(OEt)$_2$)$_2$ BN (**4a**); 161.9 MHz/CDCl$_3$; δ (ppm).

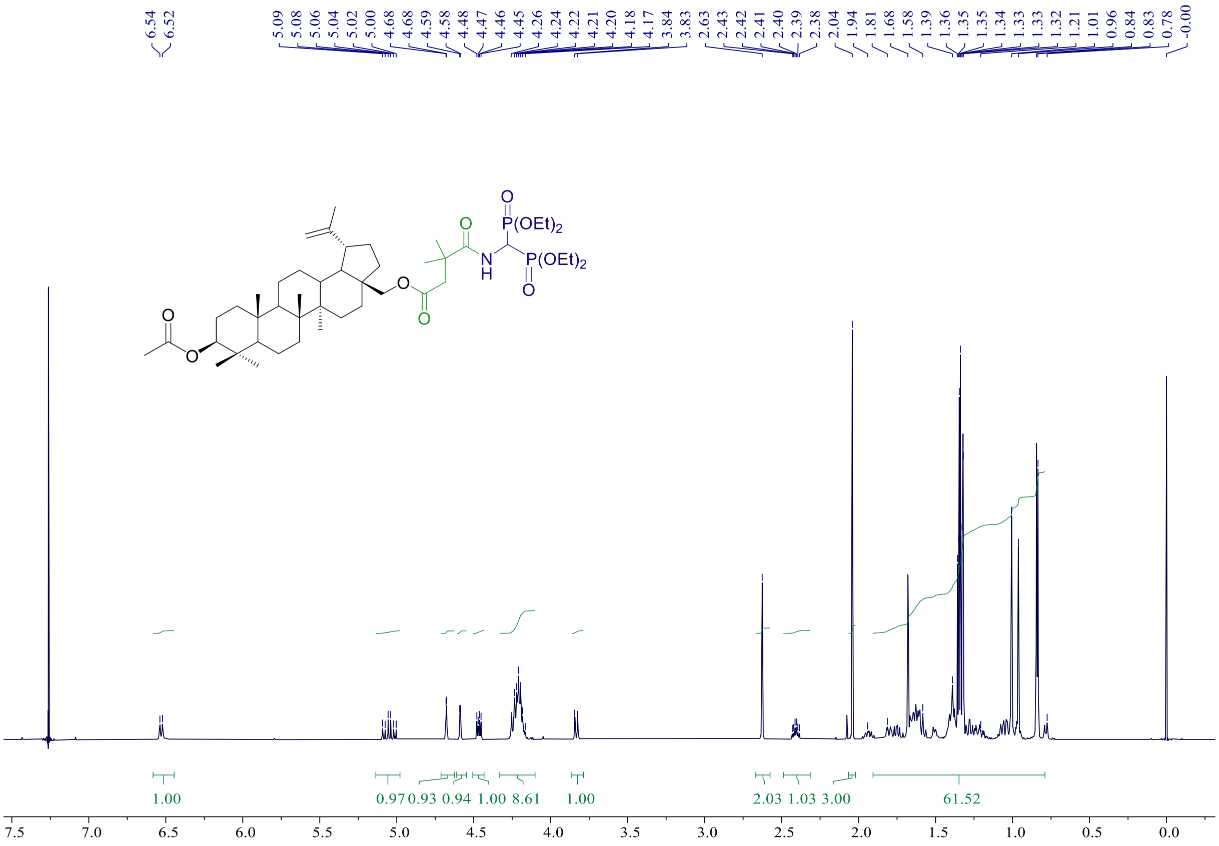

^{1}H NMR spectrum of 3-OAc-28-DMSANHCH(PO(OEt)$_2$)$_2$ BN **(4b)**; 600 MHz/CDCl$_3$/TMS; δ (ppm).

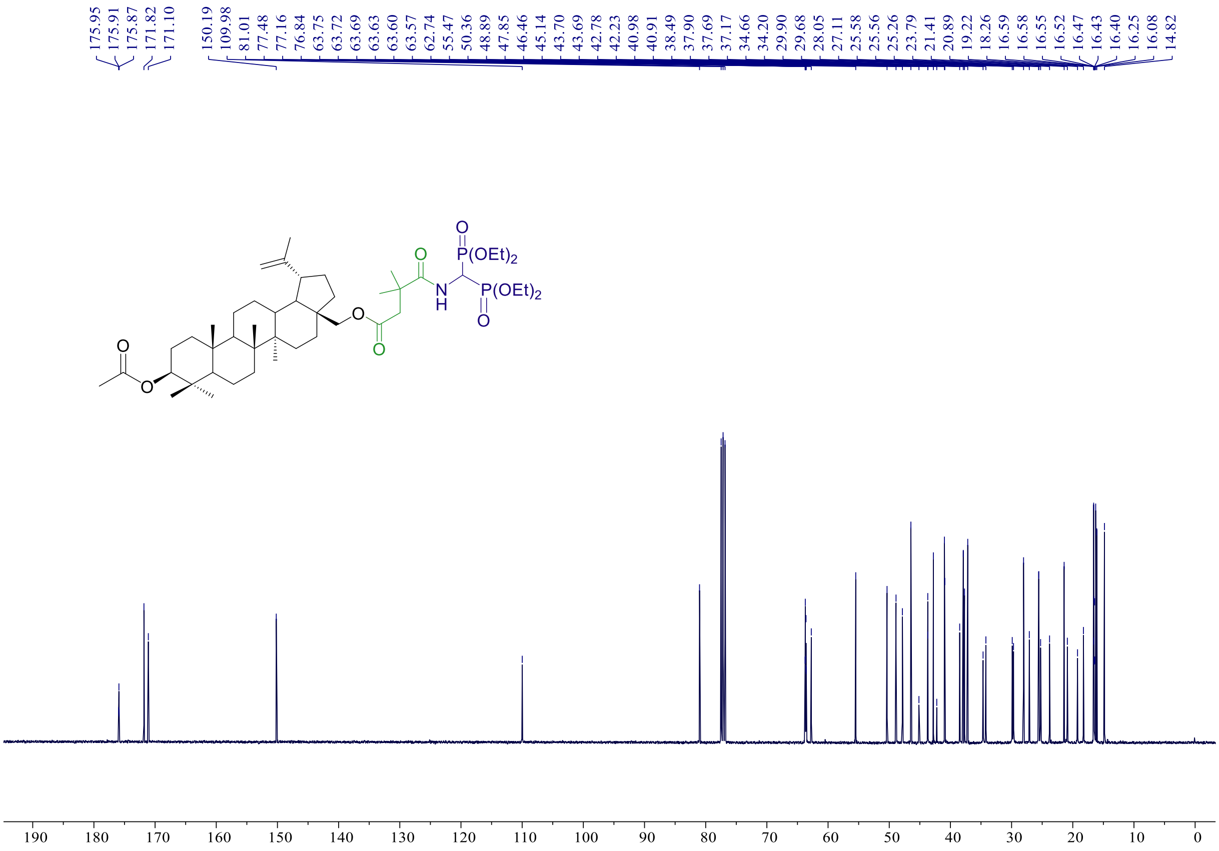

^{13}C NMR spectrum of 3-OAc-28-DMSANHCH(PO(OEt)$_2$)$_2$ BN **(4b)**; 100 MHz/$CDCl_3$/TMS; δ (ppm).

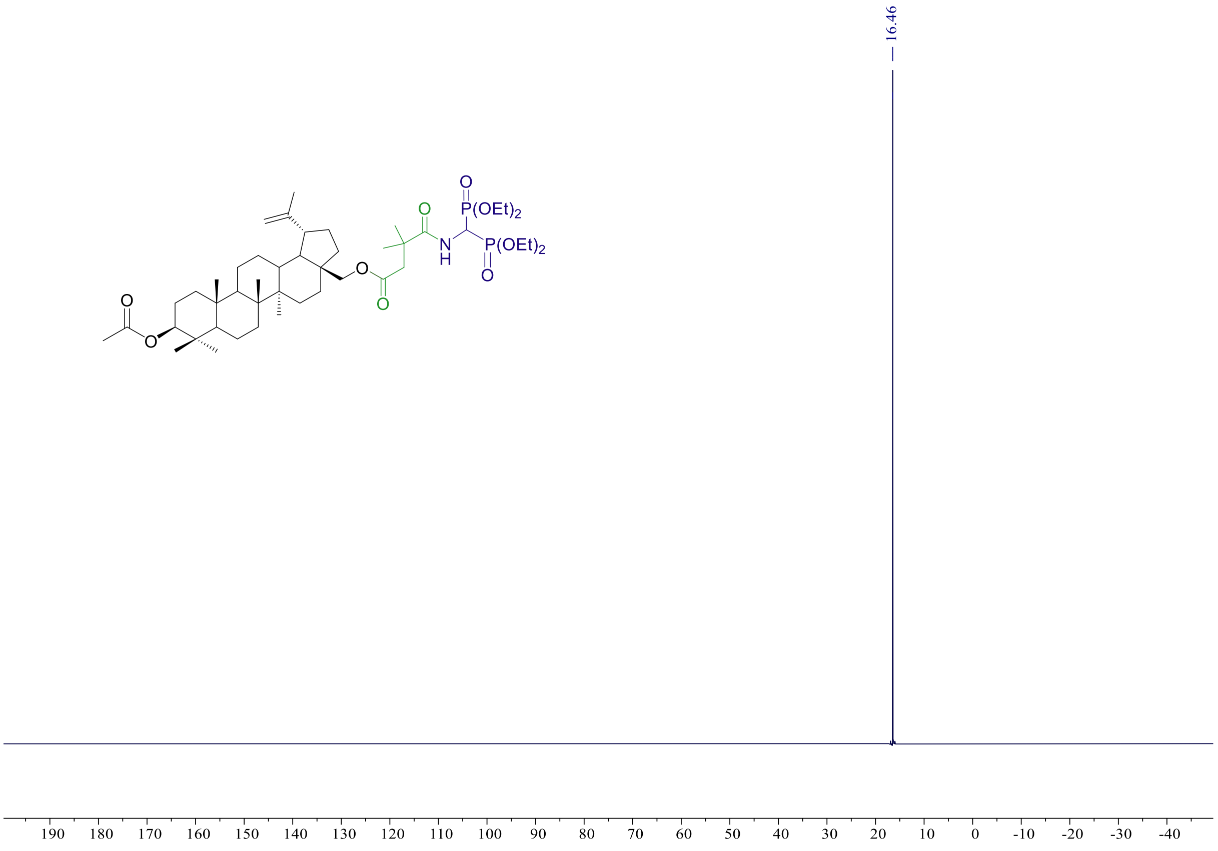

^{31}P NMR spectrum of 3-OAc-28-DMSANHCH(PO(OEt)$_2$)$_2$ BN **(4b)**; 161.9 MHz/$CDCl_3$; δ (ppm).

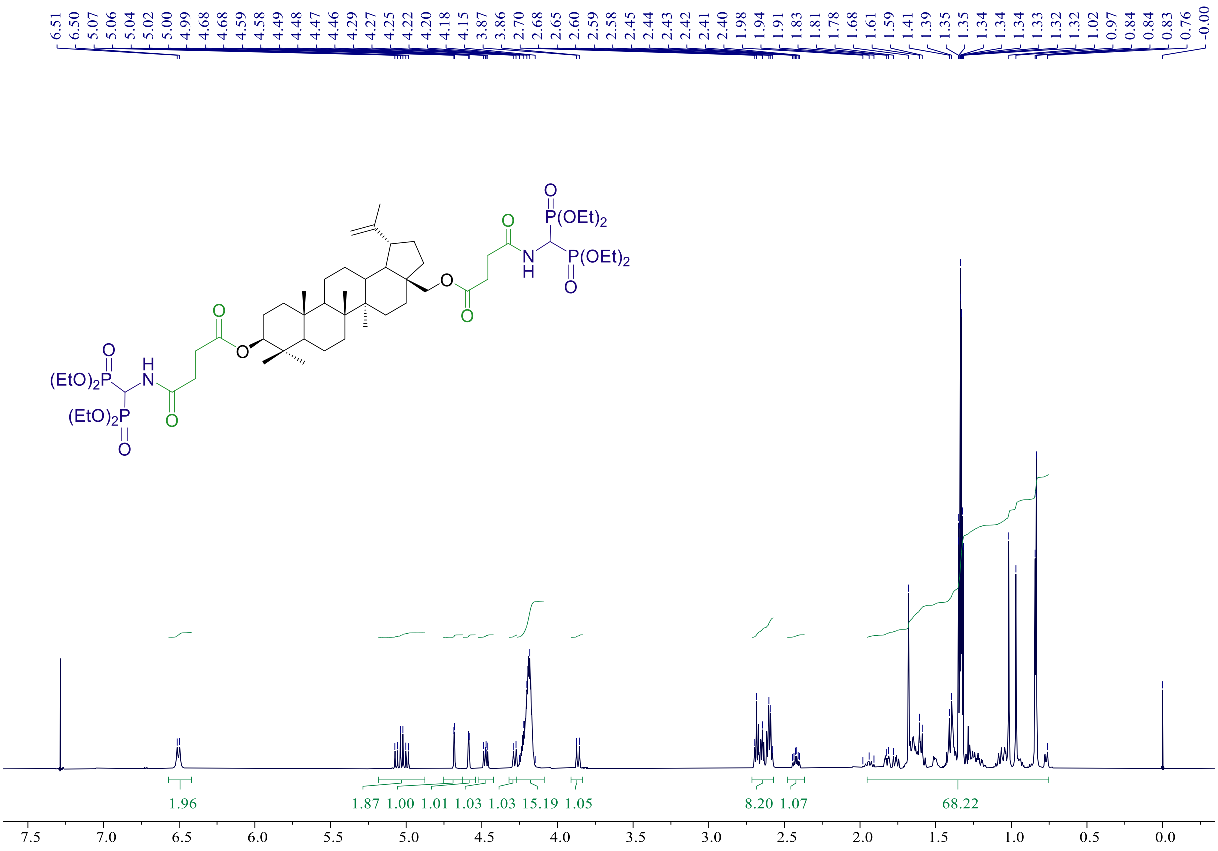

^{1}H NMR spectrum of 3,28-bis(SANHCH(PO(OEt)$_2$)$_2$) BN **(6)**; 600 MHz/CDCl$_3$/TMS; δ (ppm).

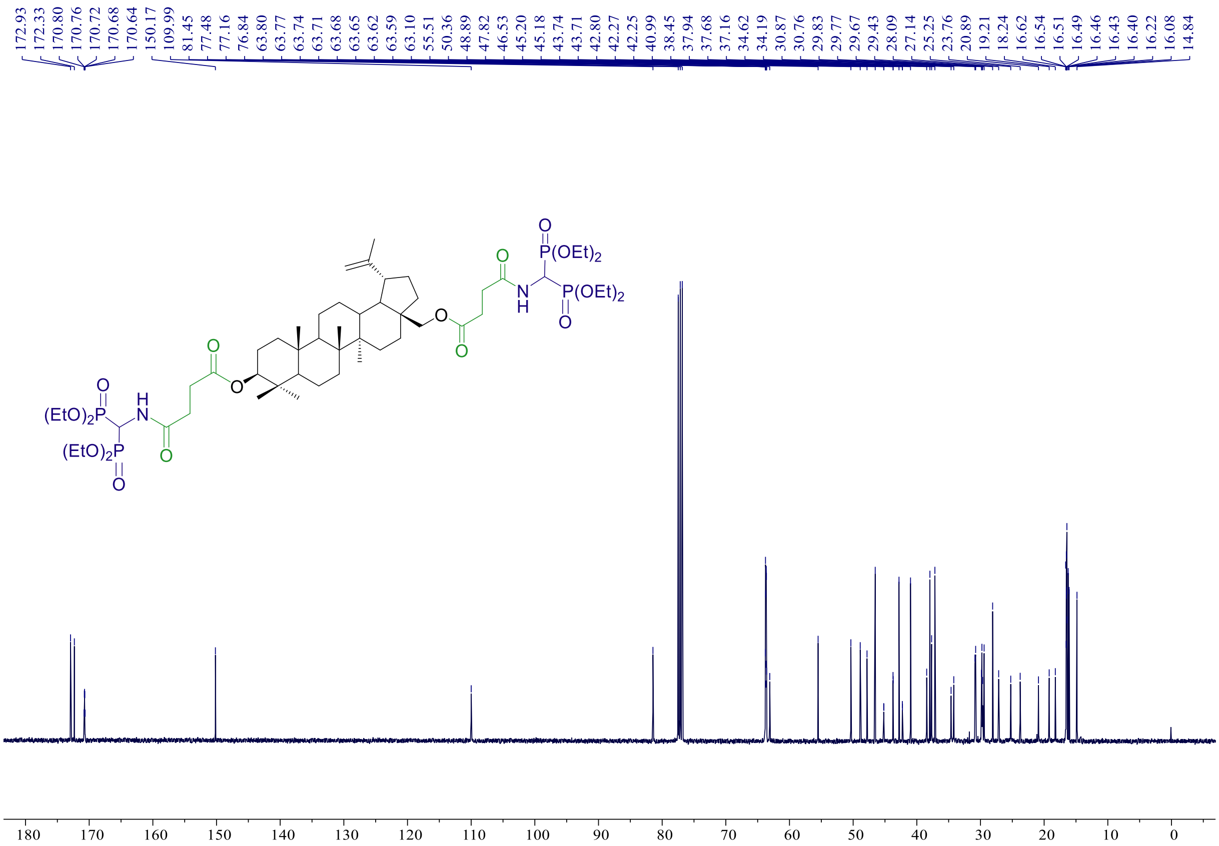

^{13}C NMR spectrum of 3,28-bis(SANHCH(PO(OEt)$_2$)$_2$) BN **(6)**; 100 MHz/CDCl$_3$/TMS; δ (ppm).

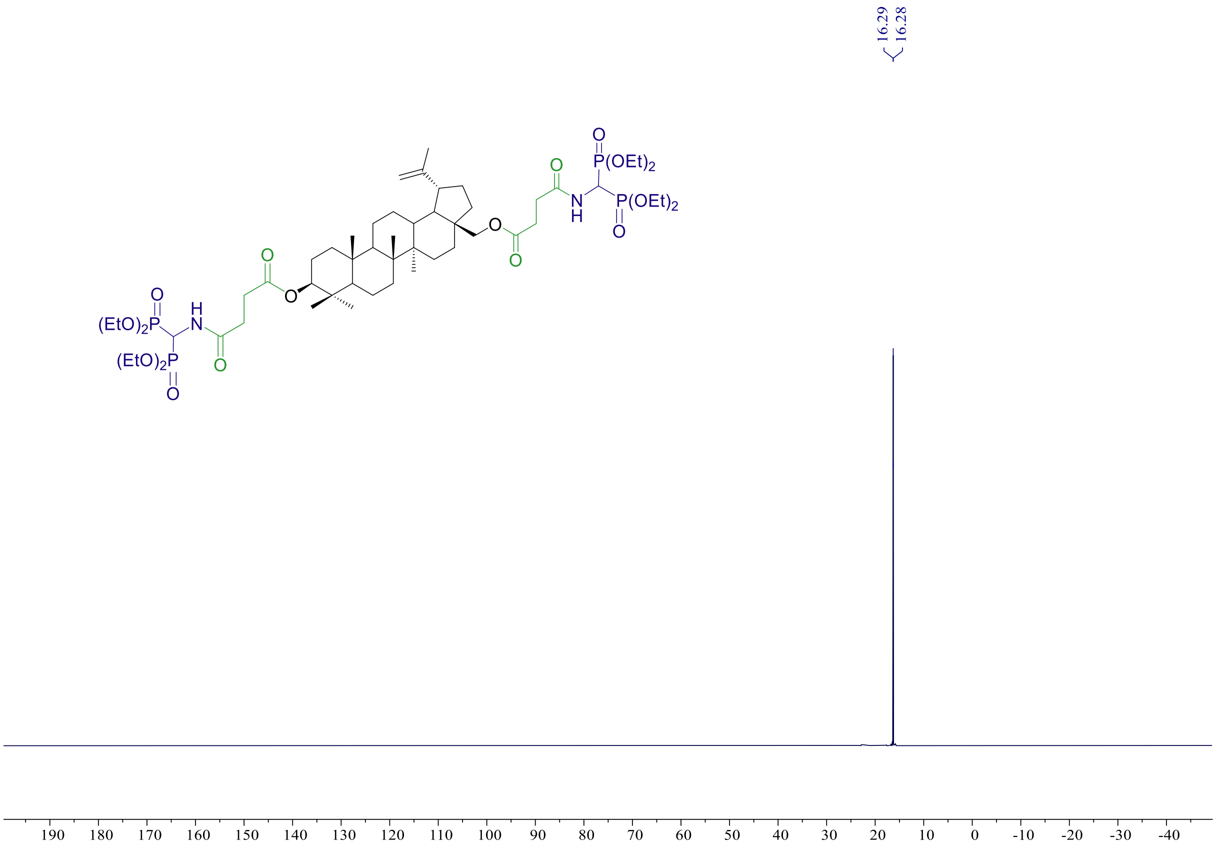

^{31}P NMR spectrum of 3,28-bis(SANHCH(PO(OEt)$_2$)$_2$) BN **(6)**; 161.9 MHz/CDCl$_3$; δ (ppm).

5. References

1. M. Grymel, A. Lalik, A. Kazek-Kęsik, M. Szewczyk, P. Grabiec, K. Erfurt, Design, Synthesis and Preliminary Evaluation of the Cytotoxicity and Antibacterial Activity of Novel Triphenylphosphonium Derivatives of Betulin, *Molecules,* 2022, **27**, 5156.

2. A. Kuźnik, D. Kozicka, W. Hawranek, K. Socha, K. Erfurt, One-Pot and Catalyst-Free Transformation of *N*-Protected 1-Amino-1-Ethoxyalkylphosphonates into Bisphosphonic Analogs of Protein and Non-Protein α-Amino Acids, *Molecules,* 2022, **27**, 3571.

3. V. Kubíček, J. Rudovský, J. Kotek, P. Hermann, L. Vander Elst, R. N. Muller, Z. I. Kolar, H. Th. Wolterbeek, J. A. Peters, I. Lukeš, A Bisphosphonate Monoamide Analogue of DOTA: A Potential Agent for Bone Targeting, *J. Am. Chem. Soc.,* 2005, **127**, 16477-16485.
